# Supplementary material for: Chrysin Directing an Enhanced Solubility through the Formation of a Supramolecular Cyclodextrin–Calixarene Drug Delivery System: A Potential Strategy in Antifibrotic Diabetes Therapeutics
Source: Pharmaceuticals (Basel). 2024 Jan 12;17(1):107. doi: 10.3390/ph17010107 (PMC10819853; doi:10.3390/ph17010107)
Supplement: Supplementary file 1 [file pharmaceuticals-17-00107-s001.zip › pharmaceuticals-2767387-supplementary.pdf]

Supplemental material

Chrysin Directing an Enhanced Solubility through the Formation of a Supramolecular Cyclodextrin–Calixarene Drug Delivery System: A Potential Strategy in Antifibrotic Diabetes Therapeutics

Anca Hermenean [1,†], Eleftheria Dossi [2,†], Alex Hamilton [3], Maria Consiglia Trotta [4], Marina Russo [5,6], Caterina Claudia Lepre [4,7], Csilla Sajtos [8], Ágnes Rusznyák [8,9], Judit Váradi [10], Ildikó Bácskay [9,10], István Budai [11], Michele D'Amico [4] and Ferenc Fenyvesi [8,*]

[1] "Aurel Ardelean" Institute of Life Sciences, Vasile Goldis Western University of Arad, 86 Revolutiei, 310414 Arad, Romania; anca.hermenean@gmail.com

[2] Centre for Defence Chemistry, Cranfield University, Defence Academy of United Kingdom, Shrivenham, Swindon SN6 8LA, UK; e.dossi@cranfield.ac.uk

[3] Biomolecular Sciences Research Centre (BMRC), Department of Biosciences and Chemistry, College of Health, Wellbeing and Life Sciences, Sheffield Hallam University, Howard Street, Sheffield S1 1WB, UK; a.hamilton@shu.ac.uk

[4] Department of Experimental Medicine, University of Campania "Luigi Vanvitelli", 80138 Naples, Italy; mariaconsiglia.trotta2@unicampania.it (M.C.T.); caterinac.lepre@gmail.com (C.C.L.); michele.damico@unicampania.it (M.D.)

[5] Doctoral School of National Interest in Public Administration and Innovation for Disability and Social Inclusion, Department of Mental, Physical Health and Preventive Medicine, University of Campania "Luigi Vanvitelli", 80138 Naples, Italy; marinarusso801@gmail.com

[6] School of Pharmacology and Clinical Toxicology, University of Campania "Luigi Vanvitelli", 80138 Naples, Italy

[7] Doctoral School of Translational Medicine, University of Campania "Luigi Vanvitelli", 80138 Naples, Italy

[8] Department of Molecular and Nanopharmaceutics, Faculty of Pharmacy, University of Debrecen, Nagyerdei St. 98, H-4032 Debrecen, Hungary; sajtos.csilla22@gmail.com (C.S.); rusznyak.agnes@euipar.unideb.hu (Á.R.)

[9] Institute of Healthcare Industry, University of Debrecen, Egyetem Square 1, H-4032 Debrecen, Hungary; bacskay.ildiko@pharm.unideb.hu

[10] Department of Pharmaceutical Technology, Faculty of Pharmacy, University of Debrecen, Nagyerdei St. 98, H-4032 Debrecen, Hungary; varadi.judit@pharm.unideb.hu

[11] Faculty of Engineering, University of Debrecen, Ótemető Street 2-4, H-4028 Debrecen, Hungary; budai.istvan@eng.unideb.hu

* Correspondence: fenyvesi.ferenc@pharm.unideb.hu; Tel.: +36-52-411-717-54505

† These authors contributed equally to this work.

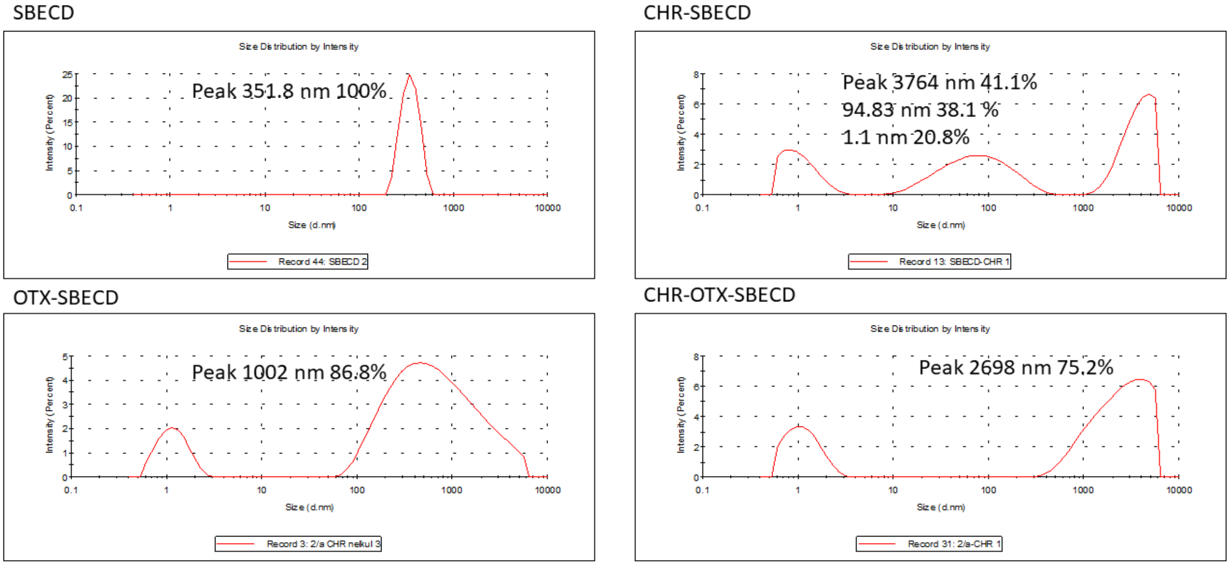

Figure S1. Size distribution of the molecular associates of SBECD, OTX008-SBECD and CHR-OTX008-SBECD in water. The associates were characterized by dinamyc light scattering. X-axis: size (nm); Y-axis: intensity distribution (%).

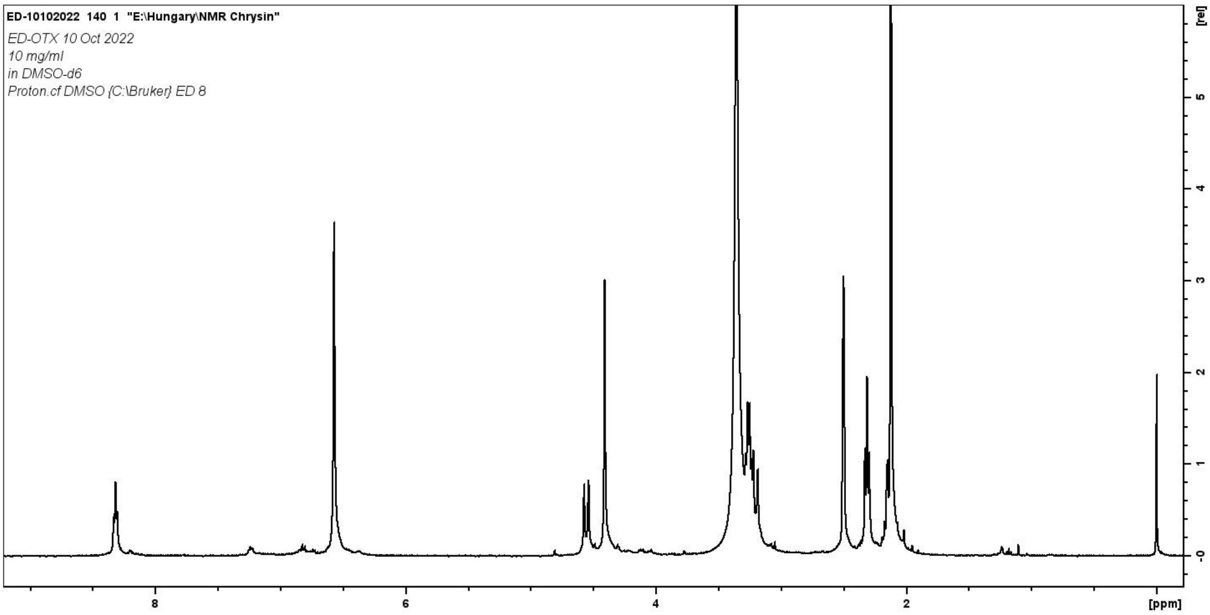

Figure S2. ^{1}H NMR spectrum of OTX008 in DMSO-d_6.

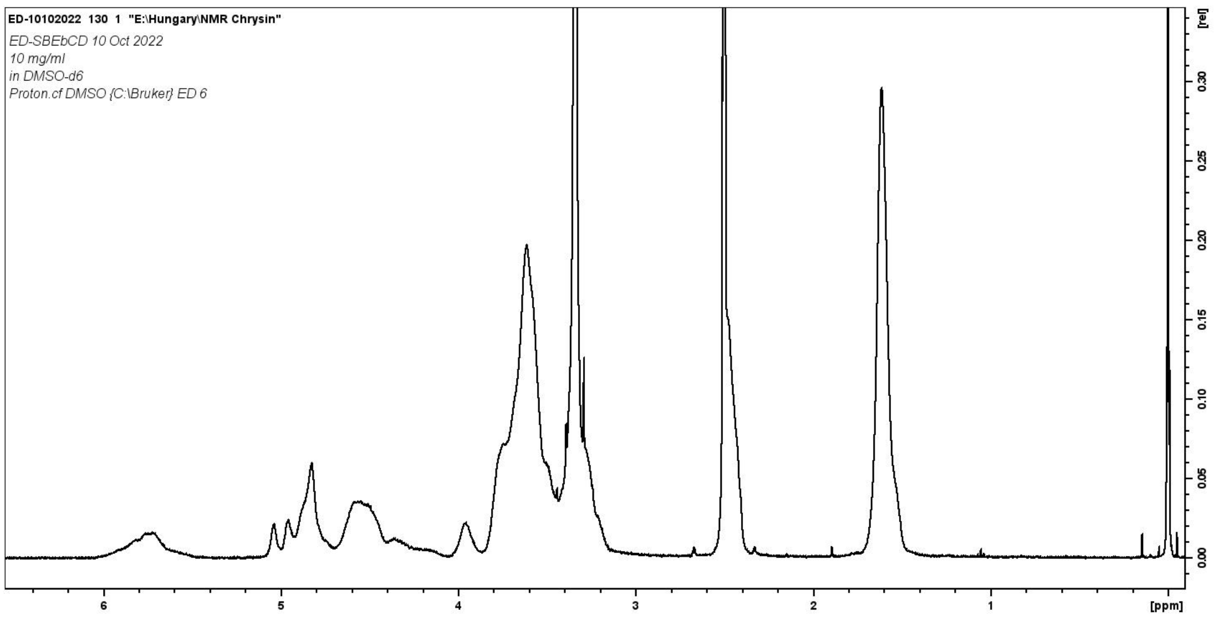

Figure S3. ^{1}H NMR spectrum of SBECD in DMSO-d_6.

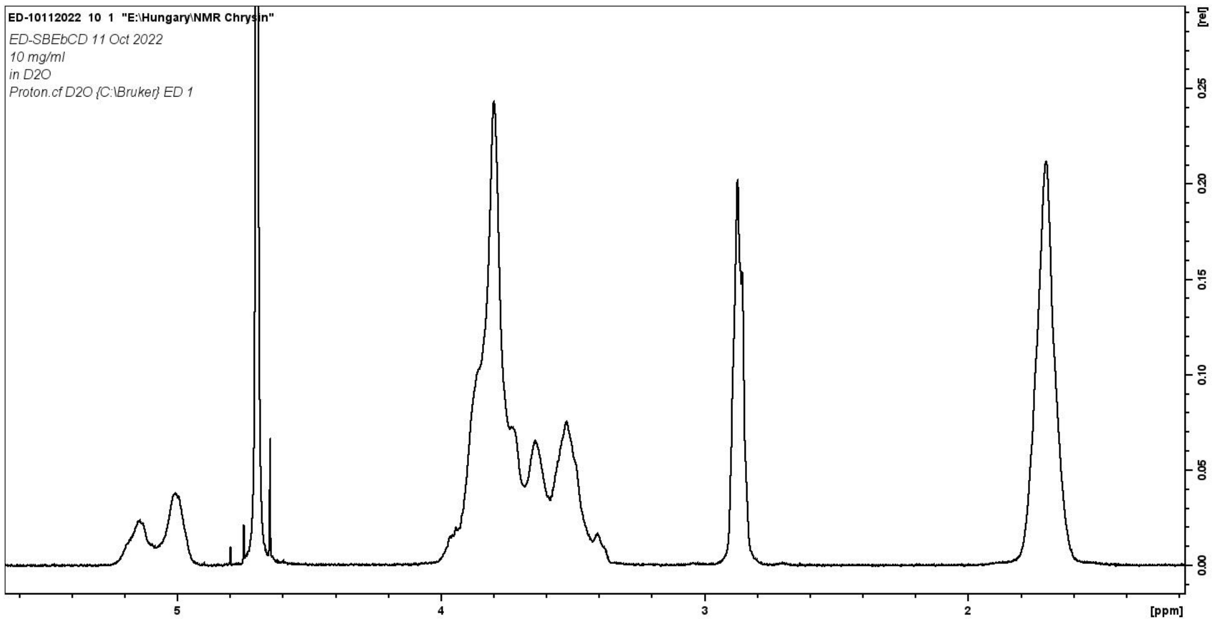

Figure S4. ^{1}H NMR spectrum of SBECD in D_2O.

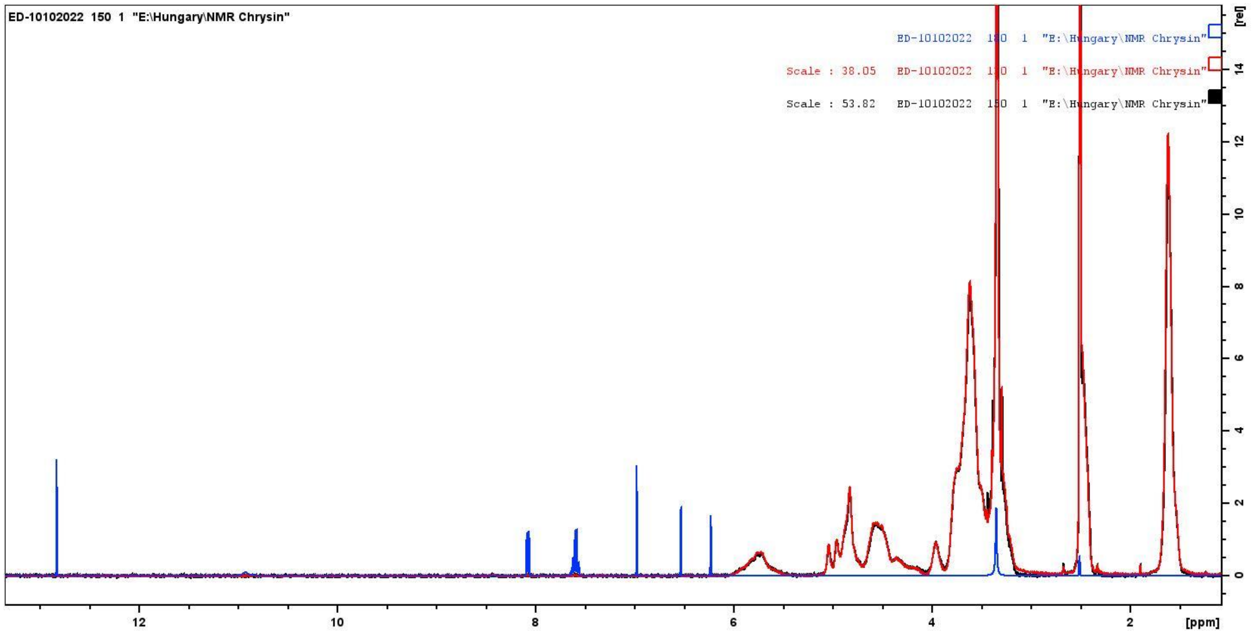

Figure S5. ^{1}H NMR spectra of CHR-SBECD mixture (black line), CHR (Blue line) and SBECD (Red line) in DMSO-d_6.

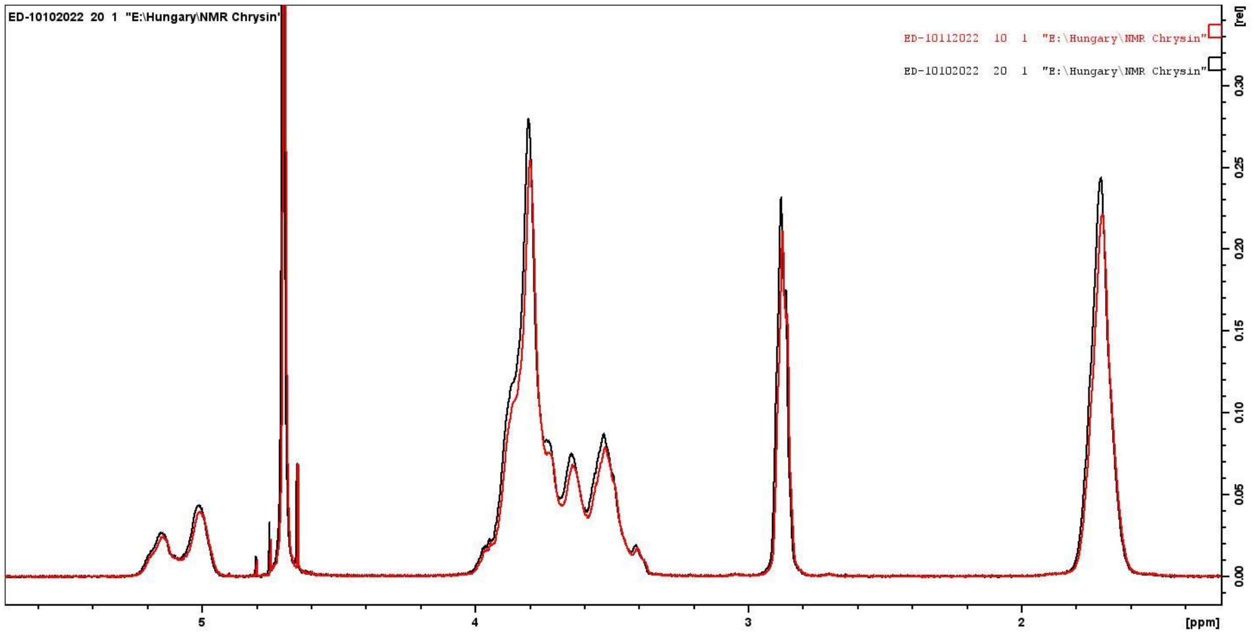

Figure S6. ^{1}H NMR spectra of CHR-SBECD mixture (black line) and SBECD (Red line) in D_2O.

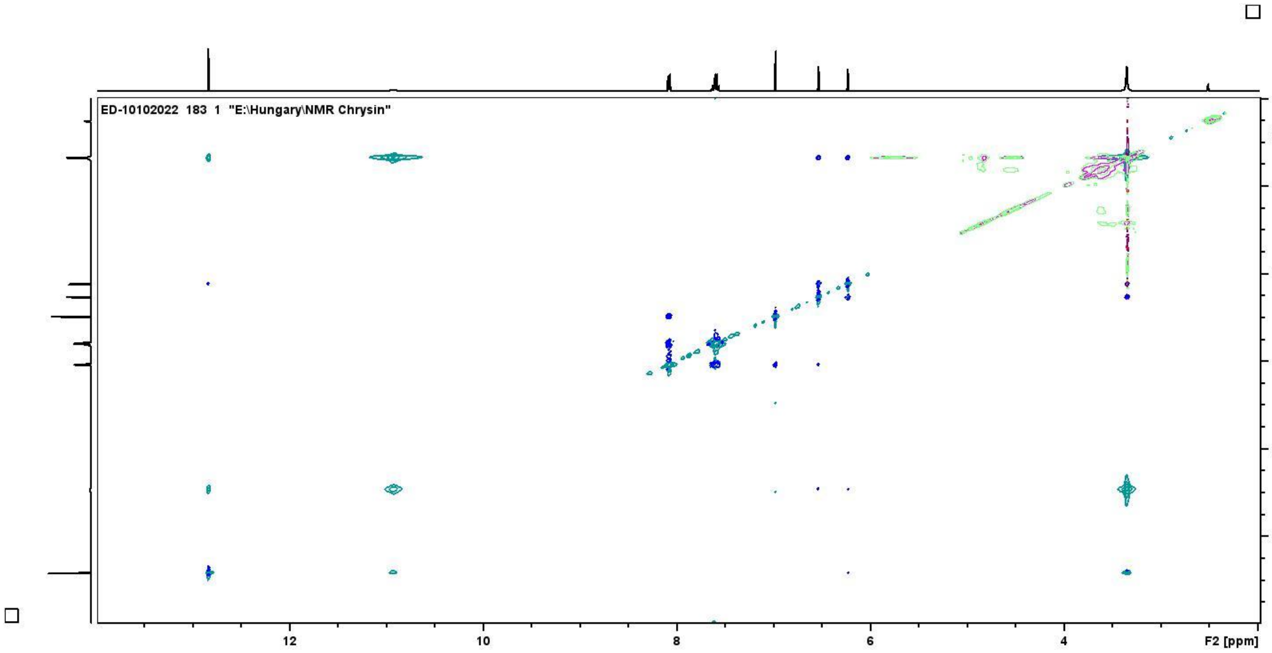

Figure S7. NOESY NMR spectra of CHR (Blue/Dark green line), CHR-SBECD mixture (Purple line) and SBECD (Light green line) in DMSO-d_6.

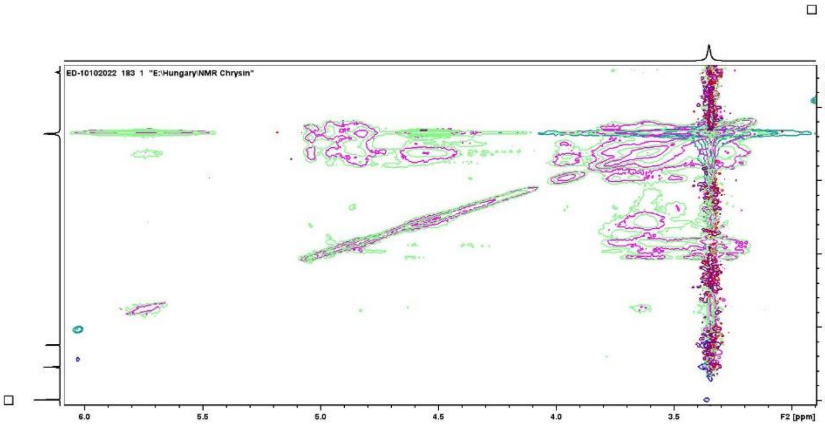

Figure S8. Expanded area of the NOESY spectra Figure S7.

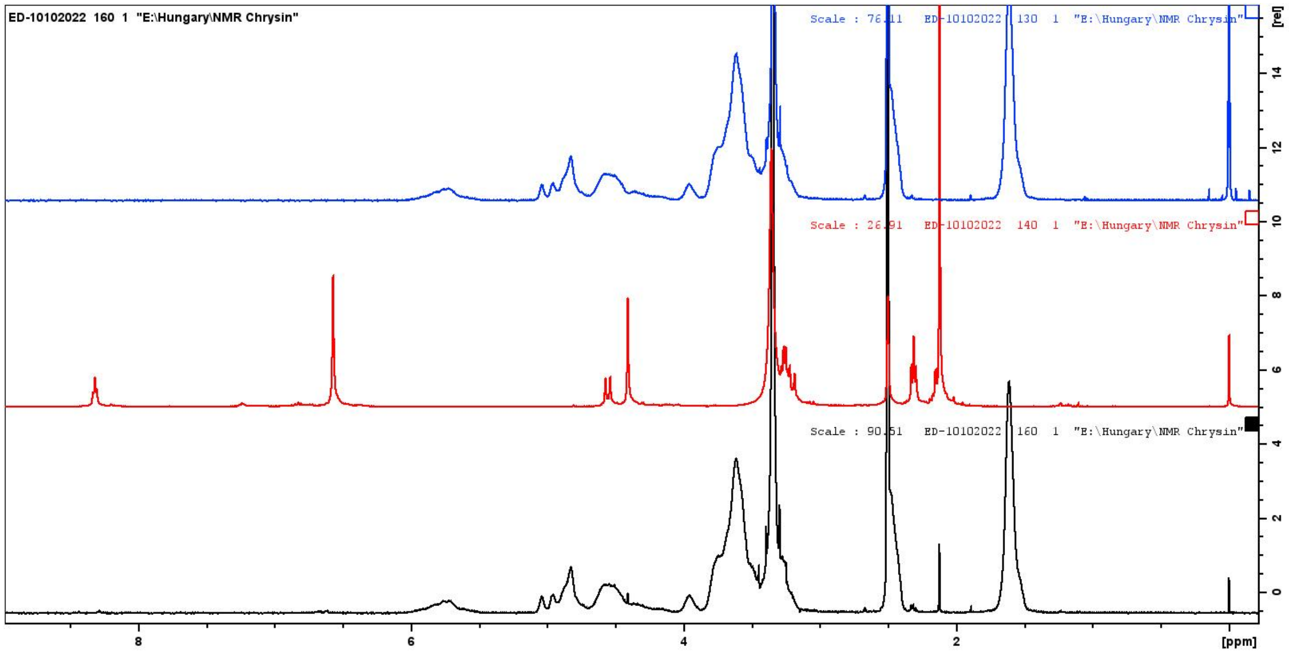

Figure S9. ^{1}H NMR spectra of OTX008-SBECD mixture (Black line), OTX008 (Red line) and SBECD (Blue line) in DMSO-d_6.

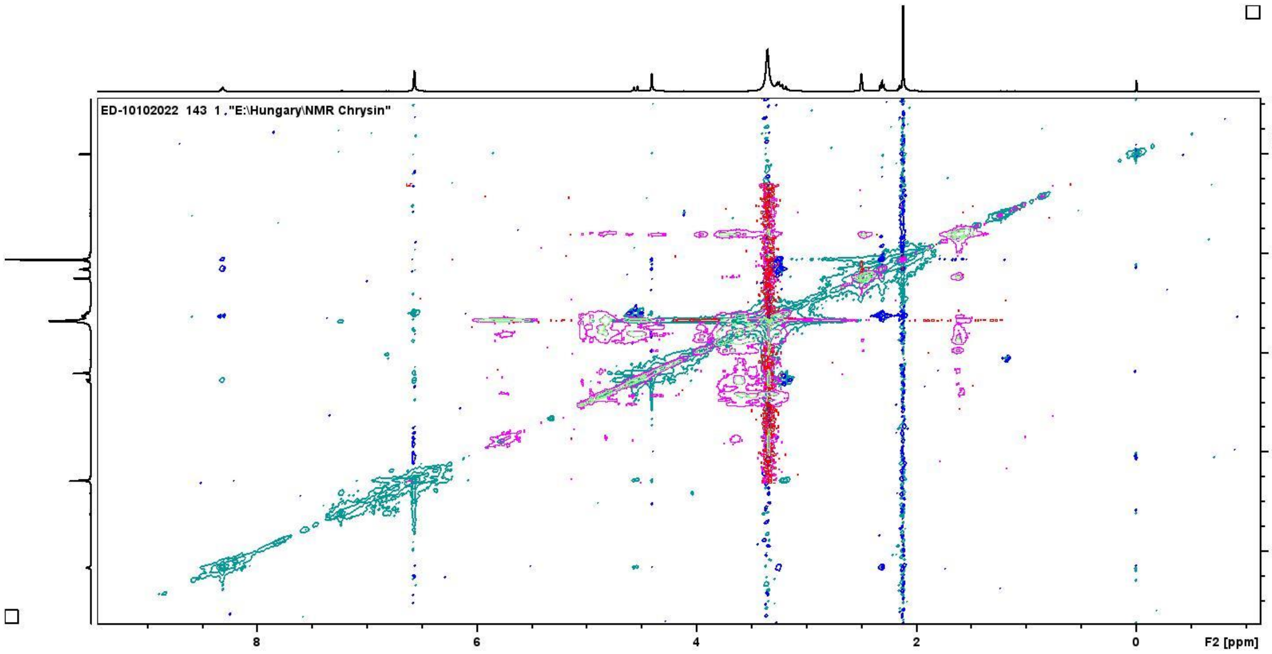

Figure S10. NOESY spectra of OTX008-SBECD mixture (Black line), OTX008 (Red line) and SBECD (Blue line) in DMSO-d_6.

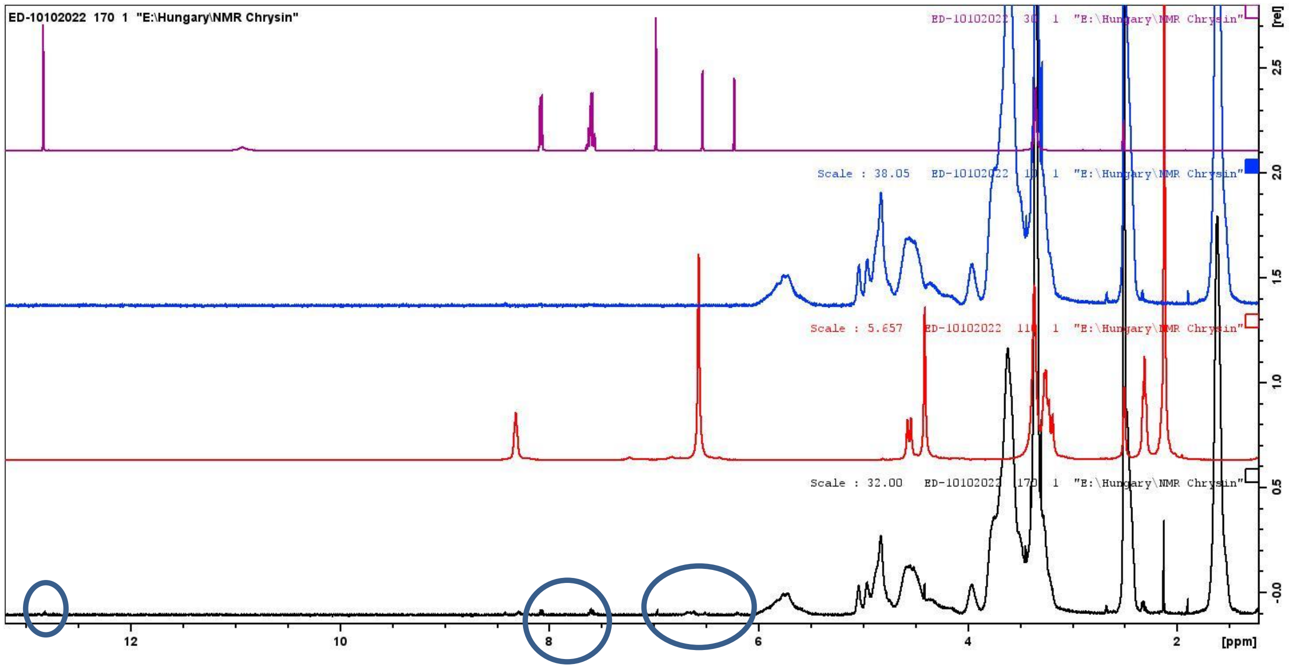

Figure S11. ^{1}H NMR spectra of CHR-OTX008-SBECD mixture (Black line), OTX008 (Red line) SBECD (B, Blue line) and CHR (Purple line) in DMSO-d_6.

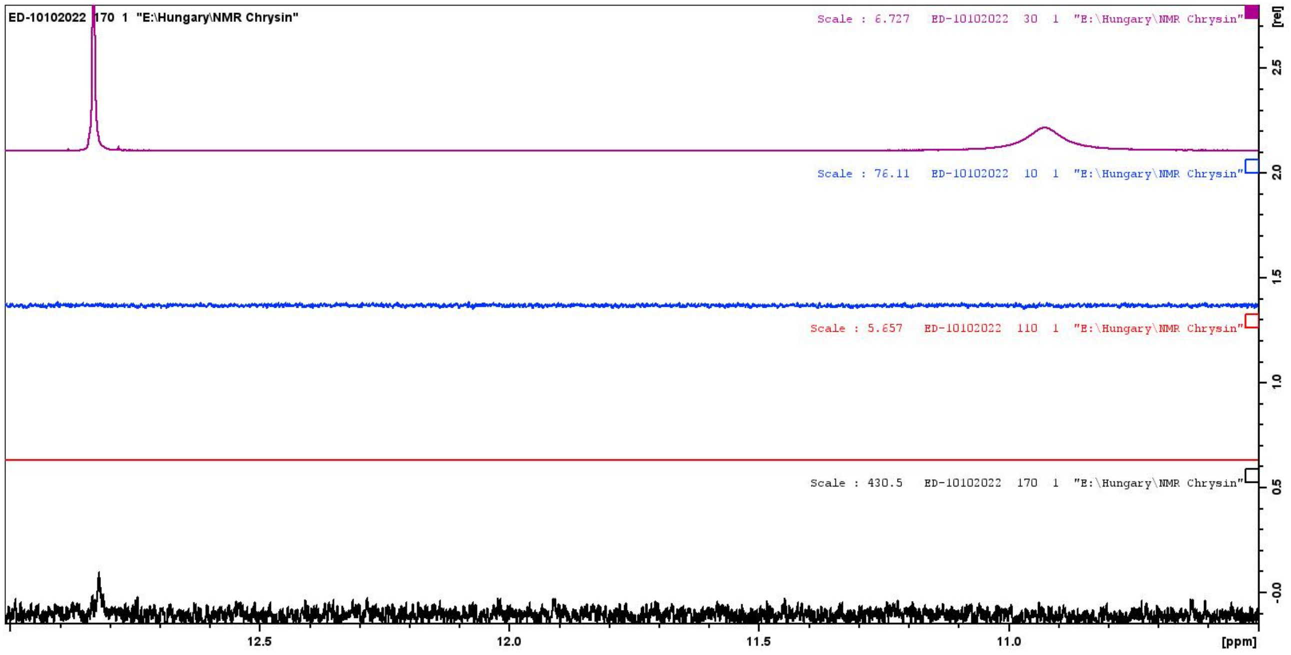

Figure S12. Expanded area of ^{1}H NMR spectra of CHR-OTX008-SBECD mixture (Black line), OTX008 (Red line) SBECD (B, Blue line) and CHR (Purple line) in DMSO-d_6.

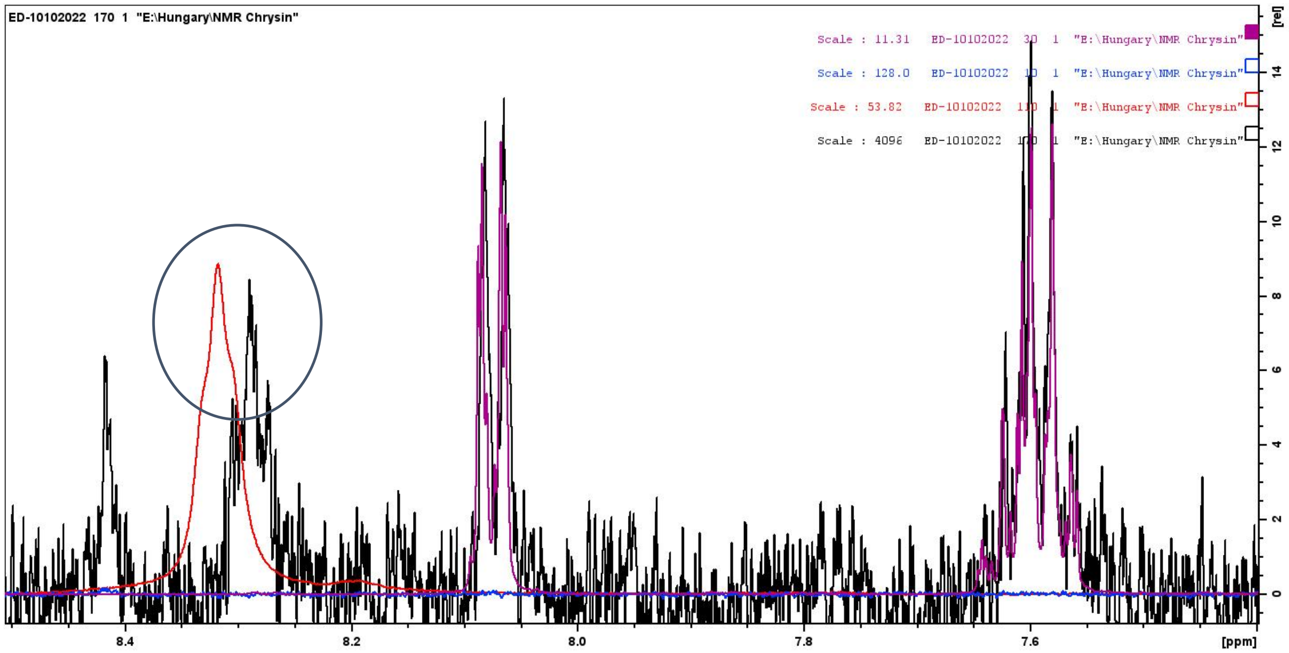

Figure S13. Expanded area of ^{1}H NMR spectra of CHR-OTX008-SBECD mixture (Black line), OTX008 (Red line) SBECD (B, Blue line) and CHR (Purple line) in DMSO-d_6.

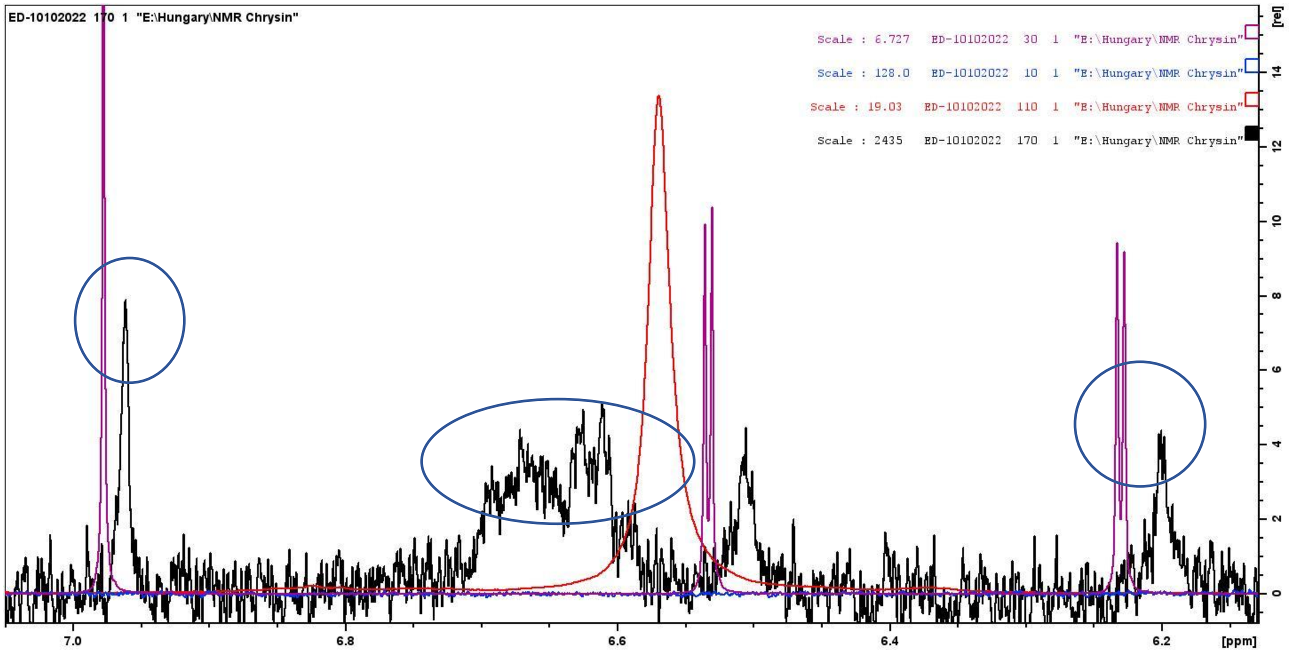

Figure S14. Expanded area of ^{1}H NMR spectra of CHR-OTX008-SBECD mixture (Black line), OTX008 (Red line) SBECD (B, Blue line) and CHR (Purple line) in DMSO-d_6.

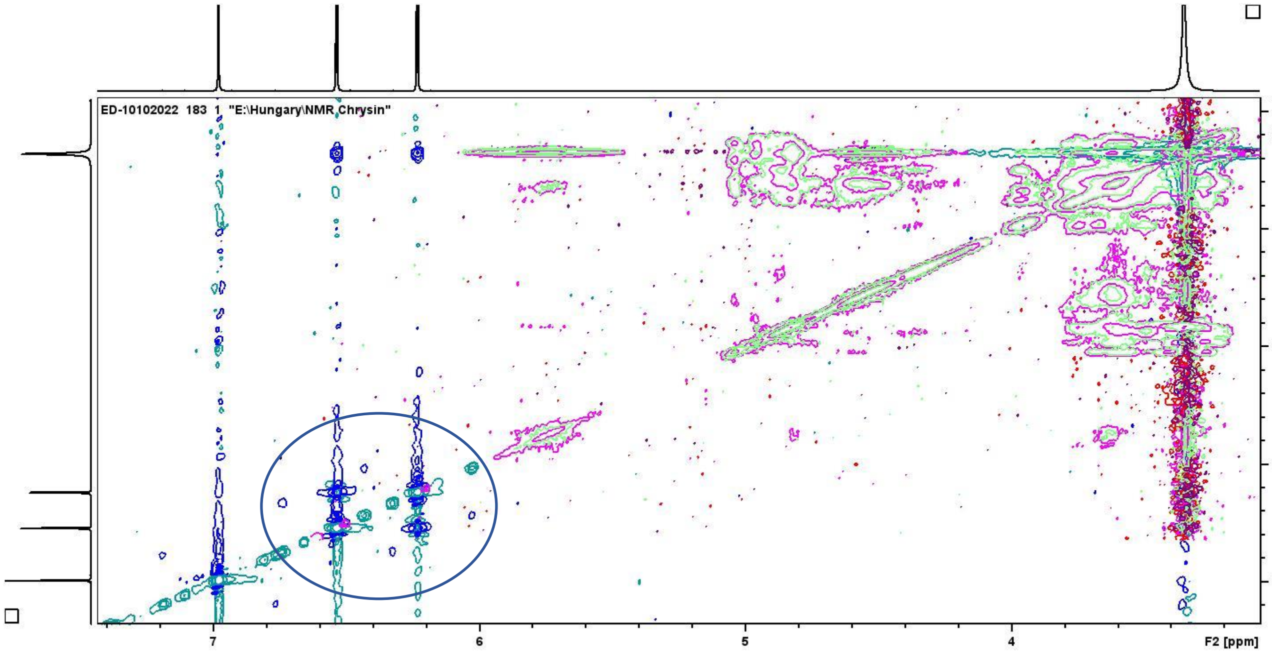

Figure S15. Comparison of NOESY spectra of CHR (Blue/Dark green line), CHR-OTX008-SBECD mixture (purple line) and binary CHR-SBECD mixture (Light green line) in DMSO-d_6. The framed parts of the spectrum shown the presense of CHR molecules in a different situation when it is in ternary mixture.

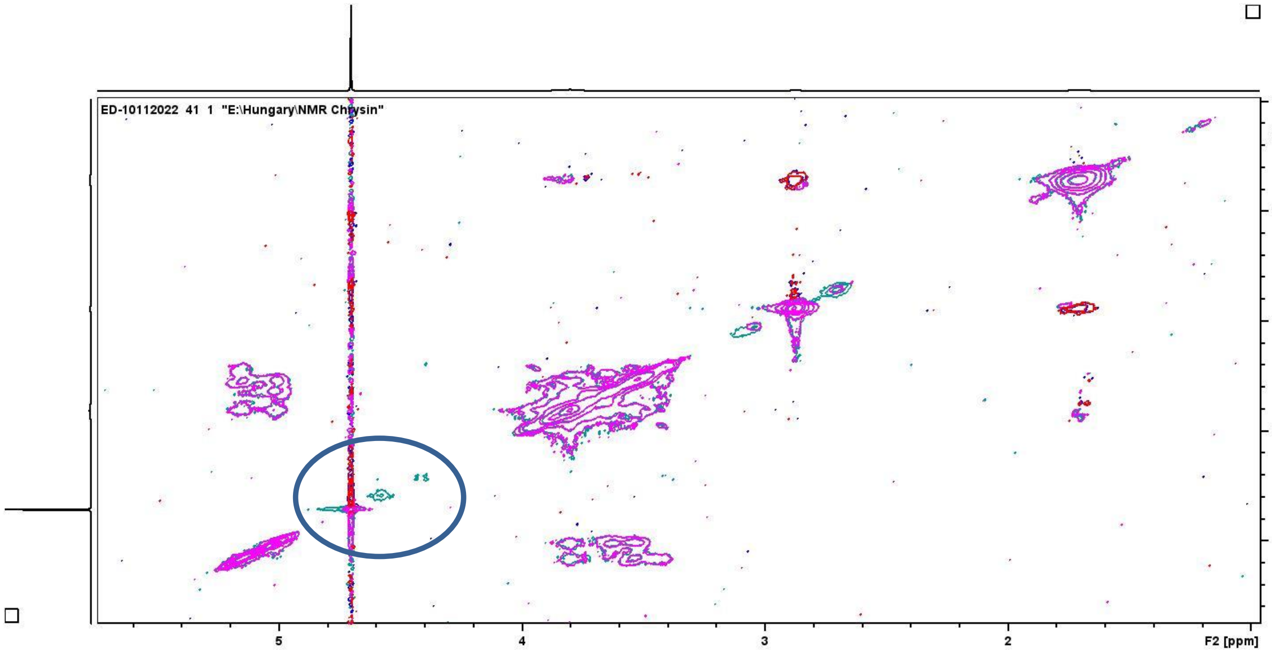

Figure S16. NOESY spectra CHR-OTX008-SBECD mixture (Black line) and SBECD (Red line) in D_2O showing the presense of CHR and OTX008 in the ternary structure.

Table S1. Results of thermal analysis.

Samples and mixtures	T_m	T_d
CHR	290	-
OTX1008	218	306
SBECD	272	281
Binary CHR- SBECD	272, 362	281
Binary OTX008- SBECD	290, 362	299
Ternary mixture<br>CHR- OTX008- SBECD	293	300

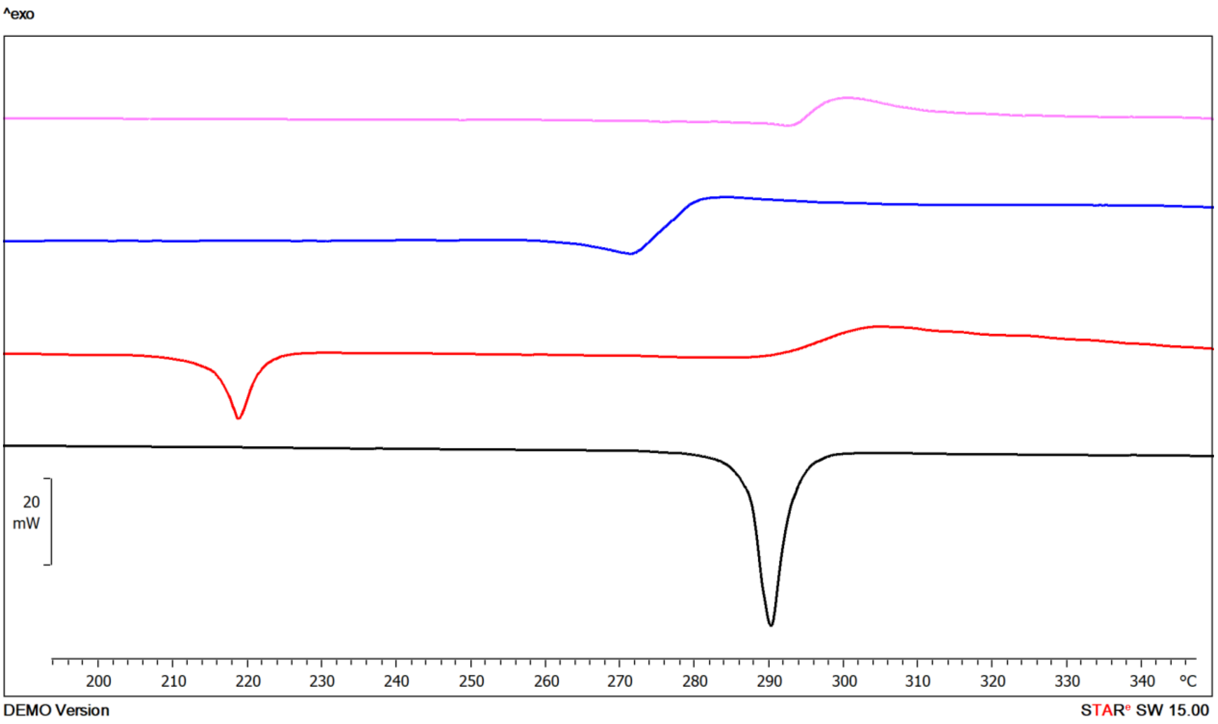

Figure S17. Zoomed view of DSC thermograms of CHR (Black line), OTX008 (Red line), SBECD (Blue line) CHR-OTX008-SBECD (Purple line), at 190-350 °C, 10 °C/min, inert N_2.

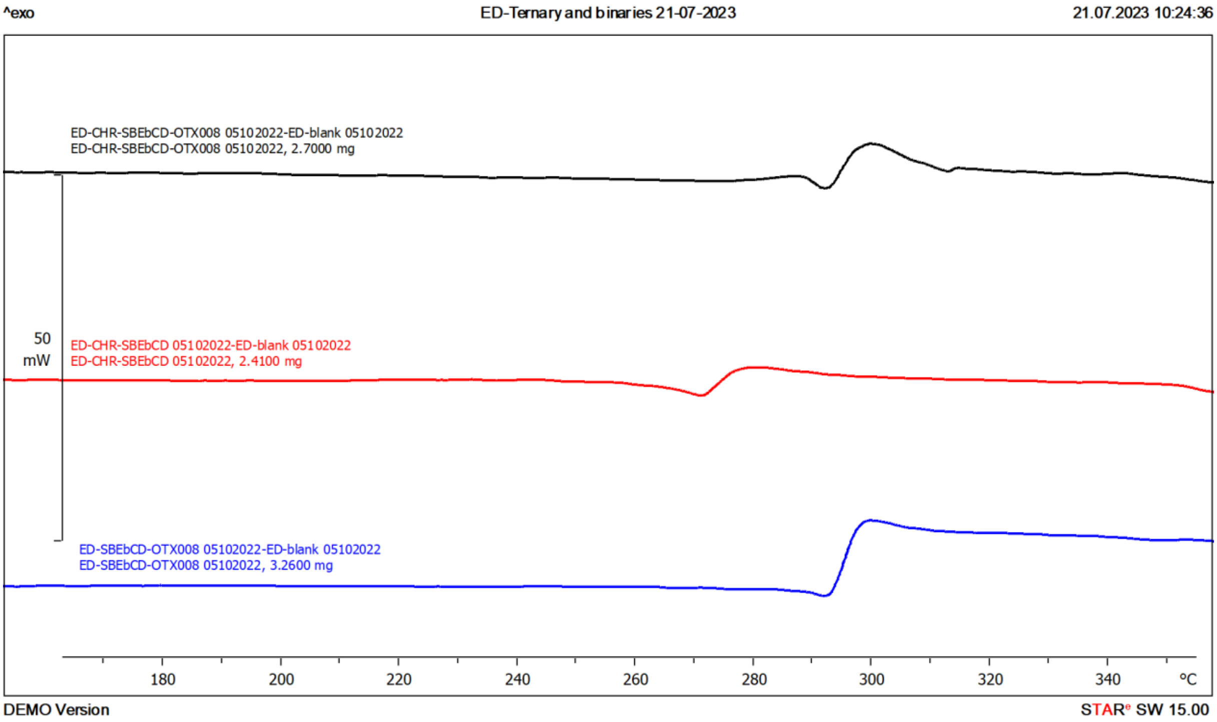

Figure S18. Comparison of zoomed areas of DSC thermograms of SBECD-OTX008 mixture (Blue line), SBECD-CHR mixture (Red line) and CHR-SBECD-OTX008 mixture (Black line), at 160-350 °C, 10 °C/min, inert N_2.

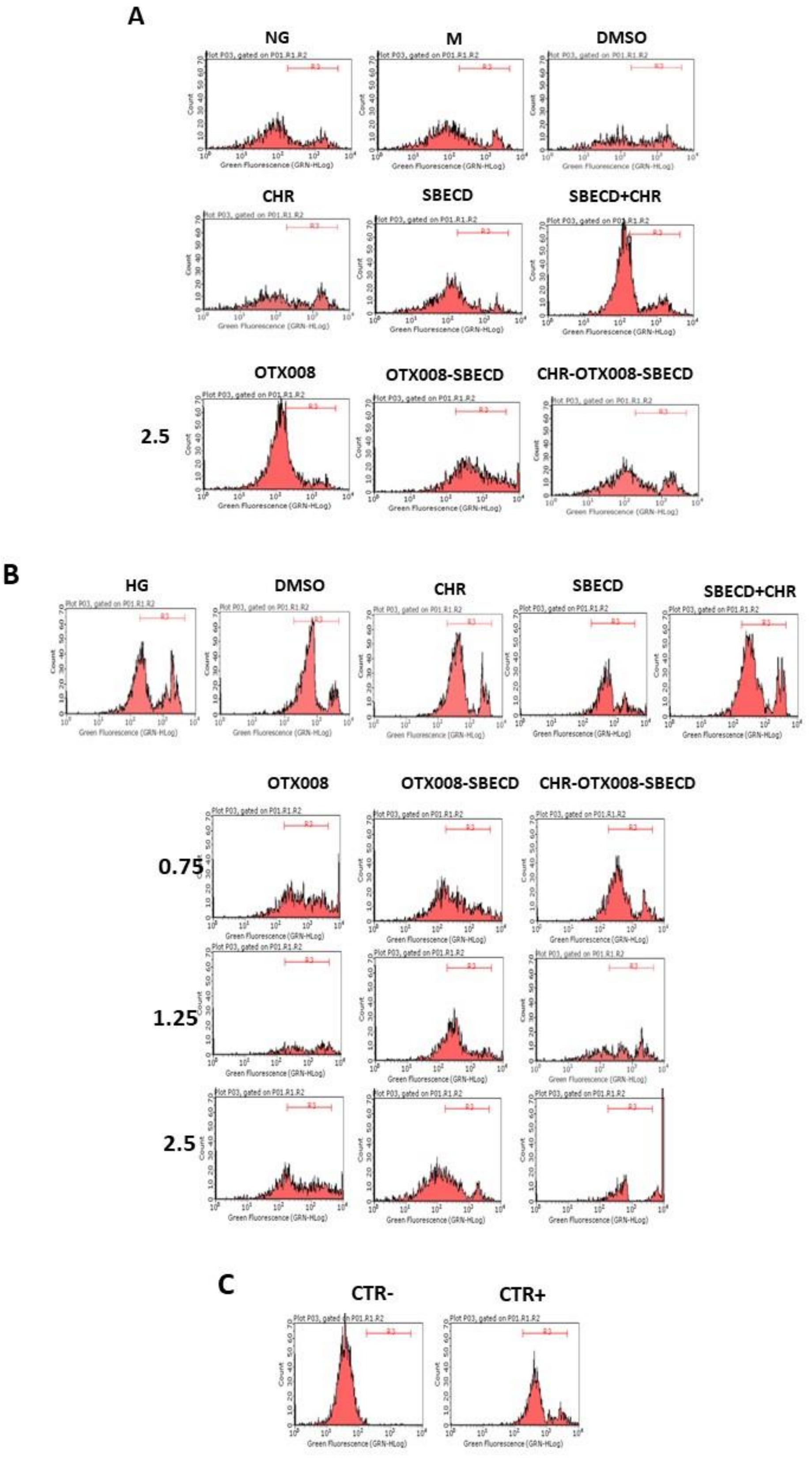

Figure S19. (A) Representative flow cytometer measures of total intracellular ROS levels assayed with DCFH-DA probe in NG or (B) HG medium. In NG cells, OTX008 was tested at

the maximum dose of 2.5 μM. In HG cells, OTX008 was tested at the doses of 2.5-1.25-0.75 μM; (C) CTR– = negative control (5% FBS without DCFH-DA); CTR+= positive control (H_2O_2 100 μM). R3 region = DCFH-DA-positive cells.

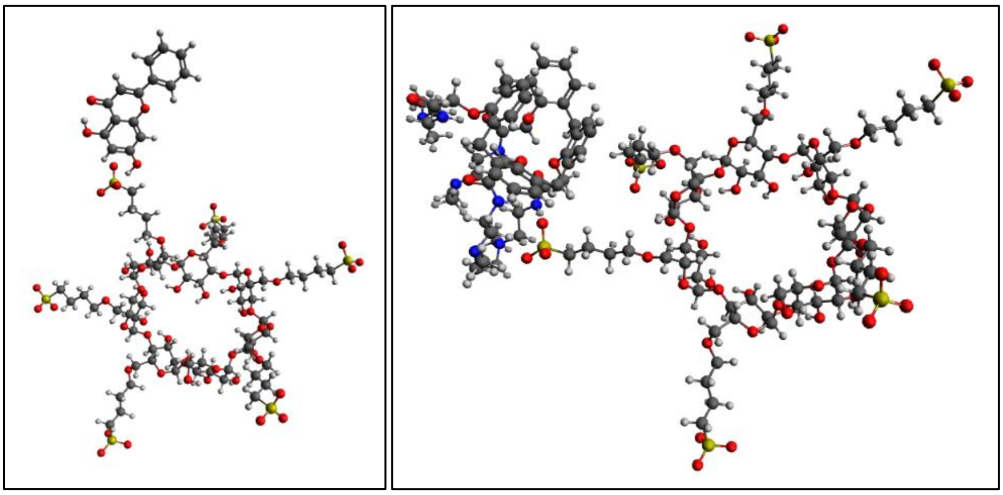

Figure S20. Interactions between SBECD and CHR (A). Interaction between OTX008 and SBECD (B).
